# Cathelicidin Antimicrobial Peptides with Reduced Activation of Toll-Like Receptor Signaling Have Potent Bactericidal Activity against Colistin-Resistant Bacteria

**DOI:** 10.1128/mBio.01418-16

**Published:** 2016-09-20

**Authors:** Cheng Kao, Xiaoyan Lin, Guanghui Yi, Yunliang Zhang, Dean A. Rowe-Magnus, Karen Bush

**Affiliations:** a Department of Molecular and Cellular Biochemistry, Indiana University, Bloomington, Indiana, USA; b Biology Department, Indiana University, Bloomington, Indiana, USA

## Abstract

The world is at the precipice of a postantibiotic era in which medical procedures and minor injuries can result in bacterial infections that are no longer effectively treated by antibiotics. Cathelicidins are peptides produced by animals to combat bacterial infections and to regulate innate immune responses. However, cathelicidins are potent activators of the inflammatory response. Cathelicidins with reduced proinflammatory activity and potent bactericidal activity in the low micromolar range against Gram-negative bacteria have been identified. Motifs in cathelicidins that impact bactericidal activity and cytotoxicity to human cells have been elucidated and used to generate peptides that have reduced activation of proinflammatory cytokine production and reduced cytotoxicity to human cells. The resultant peptides have bactericidal activities comparable to that of colistin and can kill colistin-resistant bacteria.

## IMPORTANCE

Cathelicidins are antimicrobial peptides that can also increase inflammatory responses. This combination of activities can cause complications in the treatment of bacterial infections despite the pressing need for new antimicrobials. We have identified cathelicidins with decreased activation of inflammatory responses. The peptides kill Gram-negative bacteria at low micromolar concentrations by binding to and perturbing the integrity of the bacterial membrane. The peptides were also engineered to further decrease lysis of human red blood cells. The peptides have activities comparable to those of the polymyxins, a class of antibiotics to which plasmid-borne resistance is rapidly spreading and can kill colistin-resistant bacteria. These peptides are promising candidates for the development of novel antibacterial agents.

## INTRODUCTION

More than two million people in the United States are infected by antibiotic-resistant bacteria every year (1). Overuse and misuse of antibiotics have led to a crisis where once treatable bacterial infections become deadly. The last lines of antibiotics are failing due to resistance (2–4). In late 2015, bacteria harboring plasmids that confer resistance to antibiotics of last resort, the polymyxins, were reported in China, likely due to the routine veterinary use of polymyxins (5, 6). Plasmid-mediated polymyxin resistance has since been reported in multiple countries, including the United States (6, 7). Bacteria resistant to both colistin (polymyxin E) and carbapenems, antibiotics also considered to be among those of last resort, have also been reported (8). There is an urgent need to develop novel antimicrobial agents, especially ones with complex targets that could decrease resistance.

Antimicrobial peptides are produced by organisms in all three domains of life to inhibit infection by microbes (9). Cathelicidins are a class of AMPs released from precursor proteins by proteolysis upon sensing of bacterial infection (10). The solubilized peptides are typically 25 to 45 amino acids in length with a high abundance of basic amino acids. Cathelicidins interact with the membranes of susceptible bacteria and form higher-order structures to affect membrane permeability and cause death of the bacteria (11). Cathelicidins can also bind bacterial lipopolysaccharides (LPS) that are potent inducers of inflammatory responses (12). The human cathelicidin LL-37 and the guinea pig CAP-11 can suppress sepsis in animal models (13). LL-37 and the mouse mCRAMP promote wound healing and decrease fibrosis (14–17). These properties of cathelicidins could be highly beneficial in the treatment of bacterial infection.

In addition to antimicrobial activity, LL-37 modulates innate immune signaling by the pathogen-detecting Toll-like receptors (TLRs) (18–20). LL-37 can bind nucleic acids, induce receptor-mediated endocytosis to deliver nucleic acids to endosomal TLRs, and activate proinflammatory responses (18, 20). LL-37 regulation of innate immunity and LL-37 regulation of microbial killing are both concentration-dependent activities. Normal levels of LL-37 are highly beneficial and can help prevent infections. Elevated levels of LL-37, however, are associated with autoimmune diseases such as lupus and psoriasis (21–23). The use of cathelicidins as antimicrobial agents could be limited by their proinflammatory activities.

Not all cathelicidins share the same suite of activities as those of LL-37. The mouse ortholog of LL-37 does not bind RNA and activate Toll-like receptor 3 (TLR3) signaling in mouse or human cells (20, 24). This observation suggests that, despite the fact that cathelicidins have highly similar sequences and proposed structures, they may have evolved for different activities. This possibility could be exploited to identify cathelicidins with antimicrobial activity but with limited activation of proinflammatory responses. We have identified such cathelicidins from mammals. Several cathelicidins have potent bactericidal activity and display minimal ability to activate proinflammatory responses *in vitro*. Substitutions of selected residues in the cathelicidins were found to reduce cytotoxicity to human cells. The most potent of the antimicrobial peptides has bactericidal activity comparable or better than that of colistin against Gram-negative bacteria and killed colistin-resistant bacteria.

## RESULTS

**FIG 1:**
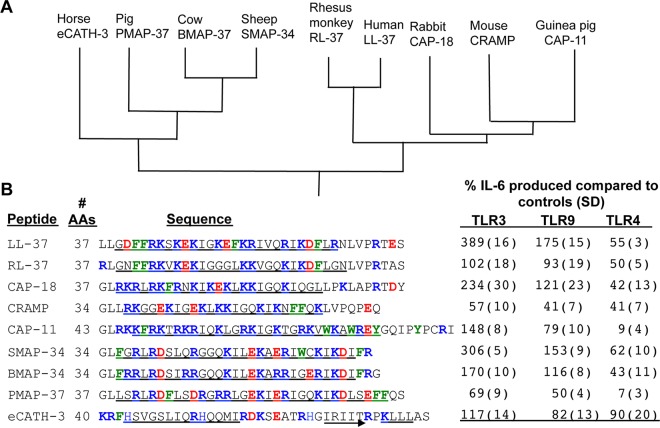
Cathelicidins from animals have distinct abilities to act on Toll-like receptor signaling. (A) A phylogenetic relationship of cathelicidins from mammals. The figure is derived from results of Zanetti et al. (12). (B) Names, lengths, and sequences of cathelicidin peptides and their ability to affect Toll-like receptor signaling. The peptide lengths are shown in the numbers of amino acids (AAs). The residues predicted to form an α-helix are underlined with an arrow. The basic, acidic, and aromatic residues are colored blue, red, and green, respectively. TLR signaling was assessed by the amount of IL-6 cytokine produced in BEAS-2B cells. IL-6 levels were quantified using enzyme-linked immunosorbent assay (ELISA), and the level of IL-6 in the control cells is normalized to 100%. The peptides were added to the cells at a final concentration of 2 μM. Each result is the mean of a minimum of three independent assays, and the standard deviations (SD) are shown in parentheses.

We sought to identify cathelicidins that can suppress inflammation induced by lipopolysaccharides (LPS) but do not activate the inflammatory response by nucleic acids. Eight cathelicidins produced by eight mammals were selected for an initial examination (Fig. 1A). The peptides were 29 to 43 amino acids in length and had a high abundance of basic amino acids. However, the number of charged residues and the lengths of the disordered residues differed (Fig. 1B). The peptides were added to BEAS-2B cells, a human lung epithelial cell line that expresses multiple TLRs and can release proinflammatory cytokines in response to their activation and the enhancement of cytokine release by LL-37 (15). BEAS-2B cells treated with the TLR3 agonist poly(I⋅C) and 2 µM LL-37 increased interleukin 6 (IL-6) production by threefold. All of the nonhuman cathelicidins tested had lower TLR3 signaling compared to LL-37 (Fig. 1B). The lack of TLR3 signaling by the nonhuman cathelicidins was due to the inability to use the formyl peptide receptor-like receptor 1 to endocytose the double-stranded RNA (dsRNA)−LL-37 complex and deliver the poly(I⋅C) to TLR3 in endosomes (17) (see Fig. S1 in the supplemental material). All of the nonhuman cathelicidins also had reduced activation of TLR9 compared to LL-37 (Fig. 1B). Activation of TLR3 and TLR9 did not correlate with the relatedness of the species from which the peptides were derived. For example, the monkey RL-37 had lower activities than that of human LL-37 while the sheep SMAP-34 had higher activities (Fig. 1B).

The nonhuman cathelicidins exhibit a range of abilities to suppress LPS-induced signaling by TLR4. LL-37 at 2 µM final concentration reduced IL-6 production to approximately half that of the untreated cells (Fig. 1B). Except for the equine eCATH-3, all of the nonhuman cathelicidins had comparable or better suppression of IL-6 production by TLR4 compared to LL-37. In summary, seven of the eight nonhuman cathelicidins had reduced abilities to activate TLR3 and TLR9 and had comparable or better suppression of the inflammatory response by TLR4.

**FIG 2:**
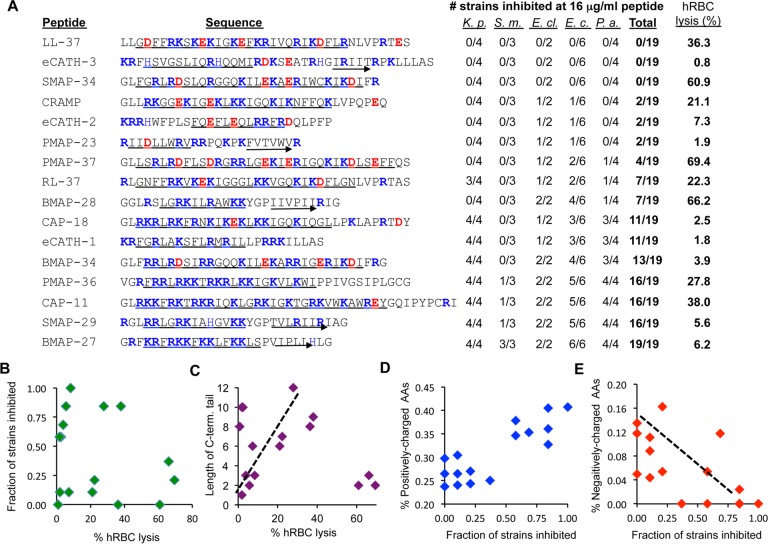
Antibacterial activity and hRBC lysis by cathelicidins. (A) Sequences of the cathelicidins, the numbers of bacterial strains killed, and hRBC lysis by the cathelicidins. The basic and acidic residues in the peptides are colored blue and red, respectively. The underlined residues are predicted to form α-helical structures. Sequences that are predicted to form a β-strand are underlined with an arrow. The bacterial species tested are *Klebsiella pneumoniae* (*K. p*.), *Serratia marcescens* (*S*. *m*.), *Enterobacter cloacae* (*E. cl*.), *Escherichia coli* (*E. c.*), and *Pseudomonas aeruginosa* (*P*. *a.*). MICs were determined by the microdilution assay. To allow comparisons of the peptides, those with MICs of 16 µg/ml or less were considered to have antibacterial activity. The results are the median values of at least three independent assays. MICs for selected peptides are shown in Table 1. hRBC lysis was assessed by the release of hemoglobin. The values shown are normalized to the amount of hemoglobin released in samples incubated in deionized water instead of phosphate-buffered saline for 1 h at room temperature. (B) Bactericidal activity of the cathelicidins does not correlate with hRBC lysis. (C) A general correlation between the length of the intrinsically disordered C-terminal tail and hRBC lysis. (D) Peptides with higher density of basic residues tend to be better at inhibiting bacterial growth. To calculate the proportion of the basic amino acid residues, arginines and lysines are counted as +1, while histidines are counted as +0.5. (E) A correlation for the presence of acidic amino acids being detrimental to antibacterial activity. Percentages are calculated as the frequency of aspartates and glutamates relative to the total length of the peptide.

The cathelicidins were examined for effects on cell proliferation. Cathelicidins added to BEAS-2B cells for 1 to 3 h all had, at most, only modest effects on cell proliferation and metabolic activity (see Fig. S2 in the supplemental material). The peptides were also assessed for lysis of human red blood cells (hRBCs). Eight of the 16 peptides tested, including LL-37, the pig PMAP-37, sheep SMAP-34, and guinea pig CAP-11, lysed more than 20% of the hRBCs (Fig. 2). The sheep SMAP-29 and the bovine BMAP-34 lysed less than 6% of the hRBCs (Fig. 2A). hRBC lysis is a convenient and sensitive method to assess the detrimental effects of the peptides.

### Antibacterial activities of the cathelicidins.

We tested a panel of cathelicidins for inhibiting growth of 19 Gram-negative strains from the family *Enterobacteriaceae* and the nonfermentative bacteria. The species tested were *Enterobacter cloacae* (2), *Escherichia coli* (5), *Klebsiella pneumoniae* (4), *Pseudomonas aeruginosa* (4), and *Serratia marcescens* (4). All of the strains selected have been characterized for their antibiotic resistance profiles. Unexpectedly, LL-37 had the least potent antibacterial activity of the cathelicidins tested, requiring at least 32 µg/ml to inhibit the growth of any of the 19 bacterial strains (Table 1). The nonhuman cathelicidins had a range of antibacterial activities. For comparative purposes, we enumerated the number of bacterial strains inhibited at 16 µg/ml (Fig. 2 and Table 1). BMAP-27 inhibited all 19 bacterial strains. With the exception of *S. marcescens*, strains were inhibited by BMAP-27 with MICs of less than 4 µg/ml. *S. marcescens* strains were more resistant, usually requiring 8 µg/ml of BMAP-27 to inhibit their growth. Notably, the antibacterial activity of the peptides did not correlate with the source of the peptides or their effects on TLR signaling.

**TABLE 1:** MICs of select antimicrobial peptides against 19 Gram-negative strains

Bacterial strain or parameter	MIC (µg/ml)																
	LL- 37	RL- 37	LL- 29	LL- 29V	LL- 29V2	CAP- 11	CAP- 11V1	CAP- 11V2	CAP- 11V3	SMAP- 29	SMAP- 29V	SMAP- 29B	SMAP- 29D	BMAP- 27	BMAP- 27A	BMAP- 27B	BMAP- 27C
*E. cloacae* 4080	32	16	16	16	16	4	4	4	8	4	4	8	4	2	2	4	2
*E. cloacae* 4092	>32	>32	>32	32	>32	8	4	16	16	4	32	16	8	2	4	4	4
*E. coli* ATCC 25922	32	16	16	8	16	4	4	8	16	4	8	8	4	2	2	4	2
*E. coli* ATCC 35218	32	8	16	16	16	8	8	8	16	4	16	32	8	2	2	4	4
*E. coli* IU0342	32	32	16	16	16	4	4	16	16	4	8	8	4	2	2	4	2
*E. coli* J53 AzideR	>32	16	32	16	32	8	4	4	16	8	8	8	8	2	4	4	2
*E. coli* MC4100	>32	>32	32	32	>32	4	8	8	16	8	8	8	8	2	4	4	2
*K. pneumoniae* ATCC 700603	>32	16	32	16	32	16	8	16	32	8	16	16	16	2	4	4	4
*K. pneumoniae* C2	>32	16	>32	16	32	8	8	8	32	8	16	8	8	2	4	4	2
*K. pneumoniae* 4110	>32	>32	>32	16	>32	8	8	8	32	16	32	8	8	4	4	8	4
*K. pneumoniae* OC8893	>32	>32	>32	16	32	4	4	8	>32	16	16	8	8	4	4	8	4
*P. aeruginosa* ATCC 27853	>32	32	>32	16	>32	4	4	8	32	8	16	8	8	2	4	4	2
*P. aeruginosa* 4083	32	16	>32	32	>32	4	4	8	32	8	8	8	8	2	4	4	4
*P. aeruginosa* PAO1	32	>32	>32	32	>32	8	2	8	32	8	16	8	8	2	4	4	4
*P. aeruginosa* PAO1 ΔOprD	32	>32	>32	32	>32	8	16	8	32	8	8	8	8	2	4	4	2
*S. marcescens* 4101	>32	>32	>32	>32	>32	>32	>32	>32	>32	>32	>32	>32	>32	16	16	16	>32
S. marcescens 4104	>32	>32	>32	>32	>32	>32	>32	>32	>32	>32	>32	>32	>32	8	8	16	16
*S. marcescens* 4075	>32	>32	>32	>32	>32	>32	>32	>32	>32	>32	>32	>32	>32	8	8	16	32
*S. marcescens* 7553	>32	>32	>32	16	>32	16	8	16	32	8	32	8	8	2	4	8	8

No. of strains inhibited at 16 µg/ml/total no. of strains	0/19	7/19	4/19	11/19	4/19	16/19	16/19	16/19	7/19	16/19	13/19	16/19	16/19	19/19	19/19	19/19	17/19

### Properties of the peptides that correlate with antimicrobial activity and hRBC lysis.

We sought to identify features of cathelicidins that correlate antibacterial activity and hRBC lysis. No correlation was observed between the percentage of hRBC lysis and the antibacterial activity of the peptides. Indeed, several peptides with high antibacterial activity had low lysis of hRBCs (Fig. 2B).

Some cathelicidins had C-terminal residues that are predicted to be intrinsically disordered. In LL-37, this tail contributes to dsRNA binding and receptor-mediated endocytosis (17). The lengths of the disordered tail did not correlate with the antimicrobial activity of the peptides (data not shown). However, with three exceptions, the peptides with longer C-terminal tails had higher hRBC lysis (Fig. 2C).

**FIG 3:**
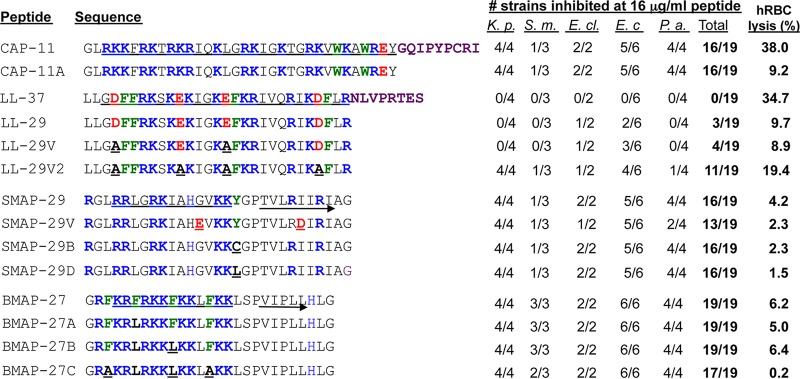
Modifications of select cathelicidins can alter their bactericidal activity, and hRBC lysis. Properties analyzed include the length of the C-terminal tail, the abundance of acidic residues, and replacements in the aromatic residues. Amino acids in the peptide sequences that were changed are shown in bold type and underlined. Sequences predicted to form an α-helix are underlined, and sequences predicted to form β-sheets are underlined with an arrow. The number of bacterial strains whose growth is inhibited at 16 µg/ml is shown. The species tested are abbreviated as in the legend to Fig. 2.

To test the effects of the C-terminal tails of cathelicidins on hRBC lysis, we removed the nine-residue C-terminal tail of CAP-11 to result in peptide CAP-11A. CAP-11A had no significant negative effects on the number of bacterial strains inhibited or the MICs (Fig. 3). Interestingly, hRBC lysis by CAP-11A was reduced from that of CAP-11. The removal of the eight amino acids that includes the intrinsically disordered C-terminal tail of LL-37, resulting in LL-29, also reduced hRBC lysis (19) (Fig. 3). LL-29 was also slightly improved in inhibiting bacterial growth compared to LL-37 (Fig. 3 and Table 1). These results suggest that the intrinsically disordered C-terminal tails in the cathelicidins contributed to hRBC lysis but not the antibacterial activity of the peptides. Consistent with this notion, two of the most effective antibacterial peptides, SMAP-29 and BMAP-27, had only two- and three-residue C-terminal tails, respectively, and relatively low hRBC lysis.

Charged amino acids in the peptides correlated with antibacterial activity. All peptides had a high percentage of basic residues. The peptides with more than 32% basic residues inhibited more bacterial strains (Fig. 2D). Another trend was that the more potent antibacterial peptides had few or no acidic residues (Fig. 2E). Indeed, acidic amino acids are underrepresented in peptides cataloged in the antimicrobial peptide database (25), and in fact, PAMP-36, SMAP-29, and BMAP-27 that all inhibited 16 or more bacterial strains lacked acidic residues. CAP-11, which also inhibited 16 of the strains, had only one acidic residue (Fig. 3). In general, peptides with higher proportions of acidic residues had reduced antibacterial activity (Fig. 3).

To examine further whether the acidic amino acids affected antibacterial activity, we made LL-29V and LL-29V2 that had two and four of the acidic amino acids in LL-29 replaced with neutral amino acids, respectively. Both LL-29V and LL-29V2 increased the number of bacterial strains killed compared to LL-29 (Fig. 3 and Table 1). Inhibition of *K. pneumoniae* was most impacted by the removal of acidic residues in LL-29.

We increased the number of acidic residues in SMAP-29 to examine the effect on the inhibition of bacterial growth, and we synthesized SMAP-29V that has two acidic residue substitutions. SMAP-29V inhibited 13 strains instead of the 16 strains inhibited by SMAP-29. The MICs for SMAP-29V for the majority of the bacterial strains also increased compared to those of SMAP-29 (Fig. 3 and Table 1). These results confirm that acidic residues in the peptides will decrease overall antibacterial activity. Altogether, these results show that the peptide sequences can be manipulated to optimize inhibition of bacteria and to reduce cytotoxicity to human cells.

### Peptides modified for reduced toxicity to mammalian cells.

SMAP-29 already had lower hRBC lysis compared to LL-37. We sought to make additional changes to SMAP-29 to further reduce hRBC lysis. Two variants that replaced the tyrosine residue within SMAP-29 with either a cysteine (SMAP-29B) or a leucine (SMAP-29D) were tested. Both SMAP-29B and SMAP-29D had reduced hRBC lysis without a significant loss of antibacterial activity (Fig. 3 and Table 1). SMAP-29B and SMAP-29D also did not affect the proliferation of BEAS-2B cells and retained low activation of TLR3 signaling (see Fig. S2 and Fig. S3 in the supplemental material). The modified SMAP-29 peptides are effective antibacterial agents for the Gram-negative bacteria tested, with reduced activation of innate immune signaling.

BMAP-27 had the most potent antibacterial activity, inhibiting all 19 of the Gram-negative strains with MICs below 16 µg/ml. It also had lower hRBC lysis than LL-37 did (Fig. 3). However, BMAP-27 reduced the proliferation of cultured mammalian cells (see Fig. S2 in the supplemental material). We sought to change the sequence of BMAP-27 to reduce the effects on proliferation of human cells. BMAP-27 has four phenylalanines within the basic residues. The antibacterial activities and hRBC lysis activities of peptides BMAP-27A, BMAP-27B, and BMAP-27C that had increasing numbers of phenylalanines replaced with nonpolar amino acids were comparable to those of BMAP-27. However, the three BMAP-27 derivatives had lower effects on cell proliferation (Fig. S2). BMAP-27B also had lowered activation of TLR3 signaling while retaining the suppression of TLR4 signaling (Fig. S3). Changes in the sequence of the peptides could therefore modulate the activities of the peptides.

### Effects on Gram-positive bacteria.

SMAP-29 and BMAP-27 and their derivatives were tested for their ability to kill Gram-positive bacteria (Table 2). SMAP-29 inhibited two strains of *Staphylococcus aureus* with an MIC of 8 µg/ml. However, with *Enterococcus faecalis*, the MIC was higher than 32 µg/ml. SMAP-29C that had a cysteine added to the C terminus of SMAP-29 had identical MICs for the four Gram-positive strains. Interestingly, SMAP-29B and SMAP-29D that each had an aromatic residue replaced by a nonpolar residue both had reduced inhibition of the Gram-positive bacteria. The derivatives of SMAP-29 and BMAP-27 series inhibited Gram-negative bacteria with lower MIC values.

**TABLE 2:** MICs of select peptides for Gram-positive bacteria

Peptide	MIC (µg/ml)			
	*E. faecalis* 51299	*E. faecalis* 29212	*S. aureus* 25923	*S. aureus* 29213
BMAP-27	>32	>32	16	16
BMAP-27A	>32	>32	16	16
BMAP-27B	>32	>32	32	32
BMAP-27C	>32	>32	>32	32
SMAP-29	32	32	8	8
SMAP-29B	>32	>32	>32	>32
SMAP-29D	32	>32	16	16

### Bacterial killing by cathelicidins.

**FIG 4:**
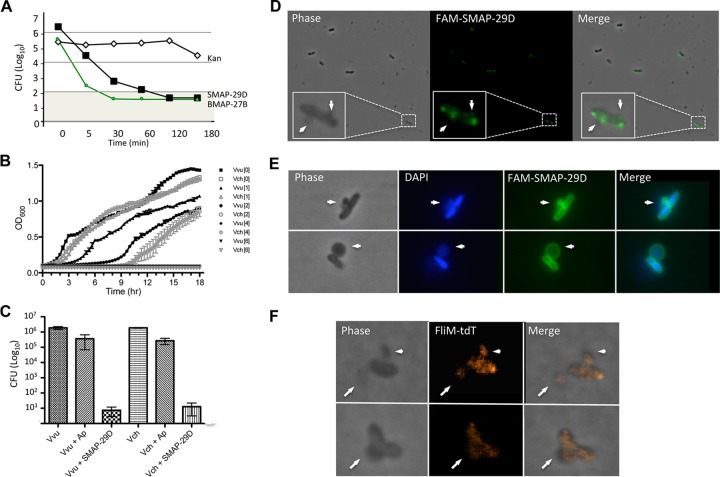
Mechanism of BMAP-27B- and SMAP-29D-induced cell death. (A) Kinetics of bacterial killing by BMAP-27B and SMAP-29D. The number of CFU of *E. cloacae* 4080 were quantified as a function of time of incubation with a 2 µM concentration of the individual peptides. In the reactions with kanamycin (Kan), 25 µg/ml of kanamycin sulfate was used. (B) Growth curves of *V. vulnificus* (Vvu) (filled symbols) and *V. cholerae* (Vch) (open symbols) in the presence of increasing concentrations (0 to 6 µM) of SMAP-29D. The initial inoculum was 10^6^ CFU in LB. (C) CFU following a 5-min incubation with 100 µM ampicillin (Ap) or 4 µM SMAP-29D. (D to F) Fluorescence microscopy of *V. vulnificus* cells. (D) Cells 30 s after treatment with 4 µM FAM-labeled SMAP-29D (green). An enlarged image of a single cell is highlighted in the boxed region. White arrows denote bulges extending from the bacterial membrane. (E) Cells 5 min after treatment with 4 µM FAM-labeled SMAP-29D (green). Bacterial DNA is stained with DAPI (blue). (F) Cells are shown 10 min after SMAP-29D treatment. The fate of cellular cytoplasmic contents was tracked following expression of a FliM-tdTomato fusion protein (red).

Cathelicidins could be either bactericidal or bacteriostatic. To distinguish between these two possibilities, we examined how BMAP-27B and SMAP-29D would affect the formation of bacterial colonies. Both peptides caused a rapid reduction in the number of CFUs. A 5-min incubation with 2 µM concentrations of the peptides resulted in a 2- to 3-log_10_-unit reduction of the viable colonies of *E. cloacae* (Fig. 4A). A 120-min incubation with 2 µM concentration of the peptide resulted in more than a 4-log_10_-unit reduction in CFU. In contrast, kanamycin resulted in a reduction of CFU only after a 2-h incubation. BMAP-27B was more effective in its bactericidal activity than SMAP-29D. Similar kinetics were observed with three *E. coli* strains, two *K. pneumoniae* strains, two *P. aeruginosa* strains, and two *Vibrio* species (see Table S1 in the supplemental material). These results demonstrate that BMAP-27B and SMAP-29D are bactericidal peptides with rapid kinetics of killing.

*Vibrio* species were used to further examine the antimicrobial activities and killing kinetics of the peptides (26). To determine whether the antimicrobial activity of SMAP-29D was bactericidal or bacteriostatic, 1 × 10^6^ CFU/ml of *Vibrio vulnificus* or *Vibrio cholerae* were inoculated into media with increasing concentrations of the peptide, and growth was monitored over 18 h. At 1 µM, both the initial growth rate and final cell density of *V. vulnificus* were significantly impacted, while *V. cholerae* appeared largely unaffected (Fig. 4B). At 2 µM peptide, the lag time to exponential growth was dramatically increased for both species, and the final number of bacterial CFU was half that of the control. Although growth was observed at extended incubation times (>7 h), these cells failed to grow when reinoculated at 10^5^ CFU into fresh media. Furthermore, growth from initial inocula of 10^6^ CFU was completely inhibited by SMAP-29D concentrations greater than 4 µM, even at 10 days postinoculation (data not shown). These results suggest that the growth observed upon extended incubation at 2 µM SMAP-29D may have been due to a small population of bacteria that escaped peptide binding rather than to the emergence of peptide-resistant mutants.

### Mechanism of bacterial killing.

To further address the kinetics of SMAP-29D activity, *V. vulnificus* and *V. cholerae* were treated for 5 min with either 100 µM ampicillin (Ap) or 4 µM SMAP-29D. Relative to control cells to which phosphate-buffered saline was added, Ap treatment decreased total CFU by less than 1 log_10_ unit, while treatment with 4 µM SMAP-29D resulted in a 5-log_10_-unit reduction in CFU (Fig. 4C). Time-lapse flow cell experiments support rapid antibacterial kinetics for the peptide. Control cells divided rapidly (16-min division time) to fully populate the chamber (see Movie S1A in the supplemental material). Over the course of 2 h, Ap-treated cells lost their characteristic curved-rod shape and began rounding up (Movie S1B). As the cell wall weakened, the bacteria ballooned to an astonishing size before eventually bursting, the consequence of a compromised peptidoglycan structure following exposure to β-lactam antibiotics. Remarkably, treatment with SMAP-29D almost immediately halted bacterial cell division, and peptide-induced membrane bulging was observed within 1 min (Movie S1C). Fluorescence microscopy with fluorophore-labeled SMAP-29D revealed that the peptide formed puncta, indicative of oligomerization on the bacterial membrane, and lesions formed at these sites (Fig. 4D). As can be seen in Fig. 4E and F, the lesions contained both DNA (stained with 4′,6′-diamidino-2-phenylindole [DAPI]) and cytoplasmic contents, which were tracked via expression of the flagellar rotor protein FliM as a TdTomato fusion (27). The DNA and cellular contents were surrounded by peptides that remained associated with outer membrane material (Fig. 4E). Eventually all of the DNA and cellular contents were displaced into the lesions (Movie S1D). The parting of internal cellular contents into a separate entity suggests that the bacterial remnants following peptide treatment were incapable of supporting further growth. Similar structures and kinetics were observed when imaging *V. cholerae* and with BMAP-27B (data not shown). These results demonstrate that SMAP-29D oligomerized upon binding to the bacterial membrane. Perturbation of the membrane led to the formation of bulged structures. Intracellular osmotic pressure caused the bacterial cytoplasm and nucleoid to fill these structures, leading to cell death. These data clearly demonstrate a rapid and potent bactericidal activity for SMAP-29D.

### BMAP-27B and SMAP-29D killing of colistin-resistant bacteria.

**FIG 5:**
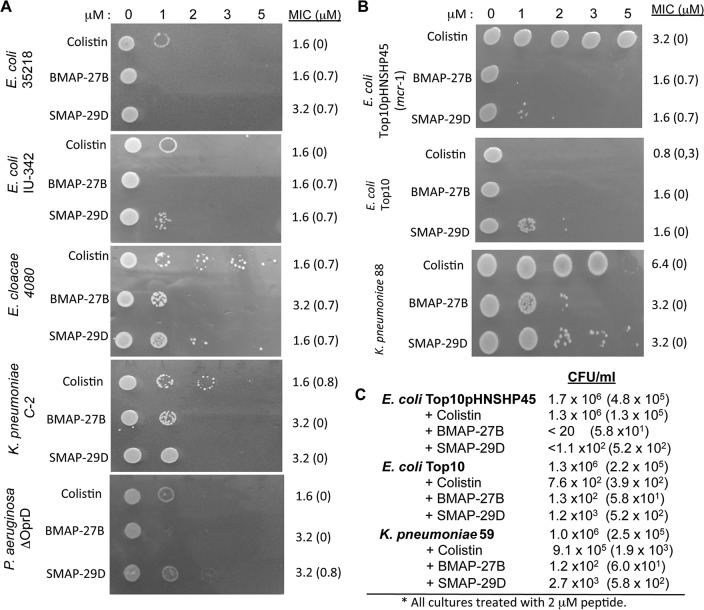
Comparison of the bactericidal activity of BMAP-27B, SMAP-29D, and colistin. (A) Colony growth of five Gram-negative bacteria treated with increasing concentrations of BMAP-27B, SMAP-29D, and colistin. A total of 1 × 10^5^ CFU of the bacteria were incubated for 30 min with the concentration of peptide or colistin shown. Approximately 500 CFU was then added as a droplet onto nonselective media for 18 h. The numbers to the right of the image of the bacterial growth were determined from a microbroth MIC determination. The number shown is the median value of three or four independent assays. The number in parentheses is the standard deviation of all of the assays. (B) BMAP-27B and SMAP-29D can kill colistin-resistant *E. coli*. The image shows bacterial growth after a 30-min incubation with colistin, BMAP-27B, or SMAP-29D. The MIC values are shown to the right of the image of bacterial growth. *E. coli* TOP10 carrying pHNSHP45 (Top10pHNSHP45) contains the plasmid-mediated *mcr*-*1* gene. TOP10 is the parental strain lacking the plasmid carrying the *mcr*-*1* gene. *K. pneumoniae* 88 is a carbapenem-resistant clinical isolate from Indiana. (C) Quantitative analysis of the viable colonies formed by colistin-resistant bacteria. *E. coli* TOP10/pHNSHP45 and the parental strain TOP10 were incubated for 5 min with either colistin, BMAP-27B, or SMAP-29D prior to plating to enumerate CFU on MH agar. *K. pneumoniae* 59 is a carbapenem-resistant clinical isolate which we found to have a MIC for colistin of 6.4 µM. The numbers are the means of three independent assays, and those in parentheses are the range for 1 standard deviation.

Recently, plasmid-mediated polymyxin resistance was found in multiple Gram-negative bacteria first in China and then in many other countries, including the United States (6, 7). We compared the bactericidal activity of BMAP-27B and SMAP-29D with that of colistin (polymyxin E). A 30-min incubation of *E. coli*, *E. cloacae*, *K. pneumoniae*, and *P. aeruginosa* with increasing concentrations of colistin, BMAP-27B, or SMAP-29D showed concentration-dependent inhibition of bacterial growth (Fig. 5A). BMAP-27B was more effective at inhibiting bacterial colony formation than colistin or SMAP-27D was. The MICs of colistin, BMAP-27B, and SMAP-29D, determined by broth microdilution assay, differed no more than twofold, although colistin often had lower MIC values (Fig. 5A). While both the plating assay and the MIC data show that BMAP-7B and SMAP-29D could kill colistin-resistant bacteria, the difference in the effective concentrations in the two assays could be due to the higher number of bacteria tested in the MIC assay, with a small subpopulation of persisting colonies. Further characterization of these persisters for cathelicidins and the frequency of resistance selection are under investigation.

Colistin resistance has been reported to confer cross-resistance to cathelicidins (28, 29). We examined whether BMAP-27B and SMAP-29D could kill the *E. coli* strain harboring the *mcr-1* gene on a plasmid named pHNSHP45 in southern China (5). *E. coli* TOP10 harboring pHNSHP45 (TOP10/pHNSHP45) (5) was resistant to colistin, as expected, but killed by 2 µM BMAP-27B and SMAP-29D with only a 5-min incubation (Fig. 5B). *E. coli* TOP10 lacking the *mcr-1* plasmid was sensitive to all three antibiotics (Fig. 5B). BMAP-27D and SMAP-29C reduced *E. coli* TOP10/pHNSHP45 colony formation by several log units with only a 5-min incubation while colistin had little effect (Fig. 5C). BMAP-27 and SMAP-29D had comparable MICs for TOP10 and TOP10/pHNSHP45 (Fig. 5B). TOP10/pHNSHP45 apparently had no cross-resistance to BMAP-27B or SMAP-29D.

We identified two *K. pneumoniae* strains that are resistant to colistin, although the basis for the resistance is not known. *K. pneumoniae* 88 (ST674) and 59 (ST258) are carbapenem-resistant clinical isolates with colistin MIC values of 6.4 µM. The MICs for BMAP-27B and SMAP-29D were 3.2 µM. In the colony formation assay, both strains had growth in the presence of 5 µM colistin (Fig. 5B and data not shown). At 2 µM, BMAP-27B and SMAP-29D reduced the number of colonies formed (Fig. 5B). Incubation with a 2 µM concentration of colistin, BMAP-27B, or SMAP-29D revealed that colistin treatment resulted in a 1.4-fold reduction in CFU, while BMAP-27B and SMAP-29D each had more than a 2-log_10_-unit reduction in CFU (Fig. 5C and data not shown). These results confirm that BMAP-27B and SMAP-29D are capable of killing colistin-resistant bacteria.

## DISCUSSION

Cathelicidins are antimicrobial peptides that can upregulate innate immune signaling. In this work, we demonstrate that cathelicidins produced by animals can have an array of activities to modulate proinflammatory TLR signaling in human cells, to suppress inflammation by bacterial ligands, and to kill bacteria. Two cathelicidins from sheep and cows were identified to have a combination of activities that hold promise to treat Gram-negative bacterial infection. BMAP-27 was also shown by Skerlava and colleagues to have potent antibacterial activities (30). Manipulation of the amino acids in these peptides resulted in peptides that, *in vitro*, caused less red blood cell lysis and had decreased effects on human cell proliferation.

Our evidence strongly supports rapid bactericidal kinetics for SMAP-29D. The peptide appears to bind to the bacterial membrane, oligomerize to form foci, and induce the formation of bulbous lesions at these puncta. The lesions fill with DNA and cytoplasmic material. This implies that the integrity of the peptidoglycan is somehow compromised in regions of peptide oligomerization. While we do not know whether the displacement of what appears to be all of the bacterium’s cellular contents into these membrane lesions requires energy or results from cellular turgor pressure alone, the outcome is the death of the bacterial cell. The *in vitro* activities of BMAP-27B and SMAP-29D against several Gram-negative bacteria were comparable to that of colistin sulfate, and these peptides could rapidly kill colistin-resistant *E. coli*.

There are several general themes for antibacterial activity of cathelicidins. First, the intrinsic disordered sequence that is commonly found at the C-terminal region of cathelicidins is not important for antibacterial activity and experimentally seemed to increase hRBC lysis. This result is consistent with those of Skerlava et al. (30), who found that the sequence C terminal to the α-helical structures of bovine cathelicidins are sufficient to kill bacteria with reduced cytotoxicity to human neutrophils and erythrocytes. Second, the clustering of basic residues is correlated with increased antibacterial activity, and the removal of acidic amino acids will further increase antibacterial activity. Third and importantly, antibacterial activity does not correlate with cytotoxicity to mammalian cells. Wang et al. (31) have also used modified amino acids in a truncated LL-37 to enhance bactericidal activity with minimal lysis of human red blood cells. Highly effective antibacterial peptides can be further modified to minimize cytotoxicity to human cells without impairment of antibacterial activity.

On the basis of their anti-inflammatory and antibacterial properties, BMAP-27B and SMAP-29D could be developed further for potential therapeutic uses. They have reduced lysis of red blood cells, likely due to decreased interactions with lipids that contain cholesterol (32, 33). The targets of the cathelicidin peptides are likely distinct from that of colistin. Bacterial mechanisms associated with resistance to LL-37 include changes in the LPS molecules, the expression of outer membrane proteases, or the use of efflux pumps to remove LL-37 (34). Colistin binds bacterial membranes through its N-terminal hydrophobic region as well as positive regions (35). Resistance conferred by *mcr*-*1* has been proposed to occur through modification of the bacterial LPS to prevent colistin binding (36). BMAP-27B and SMAP-29D likely bind bacterial membrane lipids through basic amino acids. Further characterizations of the persisters to cathelicidins and the frequency of resistance selection are under investigation. Nonetheless, difference in the mechanism of action between cathelicidins and colistin may account for BMAP-27B and SMAP-29D being able to kill colistin-resistant bacteria. Thus, based on the properties described herein, cathelicidins BMAP-27B and SMAP-29D could serve as starting points for the development of more-effective antimicrobial peptide treatments at a time when such treatments are sorely needed.

## MATERIALS AND METHODS

### Cells and reagents.

The BEAS-2B cell line was from the American Type Culture Collection and cultured in BEGM medium with its supplements (Lonza) (37). All peptides were custom synthesized by Ontores Biotechnologies (Zhejiang, China) with trifluoroacetate as the counterion. Each peptide was purified and analyzed by high-pressure liquid chromatography to have a purity of greater than 95% (Ontores). The masses and correct sequences of the peptides were analyzed using mass spectrometry. Colistin sulfate salt was from Sigma-Aldrich (St. Louis, MO). LPS was from *E. coli* O111:B4 (catalog no. L-3024; Sigma Aldrich). Poly(I⋅C) and CpG DNA ODN2006 are from InvivoGen (catalog no. 27-4732-01 and tlrl-2006, respectively) (San Diego, CA). Small interfering RNAs (siRNAs) specific to the formyl peptide receptor-like receptor 1 (sc-40123), epidermal growth factor receptor 1 (sc-29301), and nonspecific control siRNAs (sc-37007) were from Santa Cruz Biotechnology (Dallas, TX).

### IL-6 cytokine quantification.

The concentration of IL-6 secreted into the cell medium was quantified using the OptEIA kit (BD Biosciences, San Jose, CA). A typical assay used 1 × 10^4^ BEAS-2B cells/well grown for 24 h in flat-bottom 96-well plates. Poly(I⋅C) was added to a final concentration of 0.13 µg/ml for 24 h. ODN2006 was added to 0.5 nM. Lipopolysaccharides were used at 0.5 µg/ml. All data shown are the means and ranges of 1 standard error for a minimum of three independent samples. Data sets were compared using the Student *t* test calculated with GraphPad Prism 5 software.

### Peptide secondary structure analysis.

Peptide secondary structures were analyzed using the Jpred4 program (38). The intrinsically disordered residues were predicted using the program PONDR.

### MIC determination.

Antimicrobial activity was determined using the broth microdilution method based on the guidelines of the Clinical and Laboratory Standards Institute (CLSI) (39), using cation-adjusted Mueller-Hinton broth (CAMHB) as the testing medium. Reference Gram-positive and Gram-negative bacteria were tested, including both enteric and *Pseudomonas aeruginosa* strains. Each MIC was determined in at least three independent assays.

### Cell cytotoxicity assays.

The hemolytic activities of peptides were determined using human red blood cells (hRBCs) (catalog no. IPLA-WB3-18103; Innovative Research, Inc., Novi, MI). The hRBCs were washed three times with phosphate-buffered saline (PBS) (pH 7.4) and then resuspended in PBS. hRBC solution was mixed with twofold serial dilutions of peptides in PBS buffer starting with 0.5 µM to 4 µM. The reaction mixtures were incubated for 45 min at 37°C. After centrifugation at 94 × *g* for 10 min, the intact hRBCs were pelleted, and the hemoglobin released from hRBCs was monitored by measuring the absorbance of the supernatant at 415 nm. The background level of absorbance was measured in peptides incubated with only PBS buffer. hRBCs incubated with water were used as the reference for 100% hemolysis. The percentage of hemolysis was calculated according to the following equation: percentage of hemolysis = [(*A*_sample_ − *A*_blank_)/*A*_water_] × 100 where *A*_sample_, *A*_blank_, and *A*_water_ are the absorbance of the sample, blank, and water, respectively.

Cell proliferation assays were performed with the CellTiter-Glo luminescent cell viability assay (catalog no. G7572; Promega). Assay plates used cultured human BEAS-2B cells amended with peptides for 2 h. An aliquot of 100 µl of CellTiter-Glo reagent was added to each well and incubated at room temperature for 2 min. Luminescence was recorded using PerkinElmer’s Victor3 V multilabel counter. All data shown are the means and ranges for one standard error for a minimum of three independent samples, and comparisons of the results from different data sets were analyzed by the Student *t* test.

### Fluorescence microscopy.

Single-cell static images and movies were captured on an Olympus IX83 inverted microscope using a 100×, 1.3-numerical-aperture phase-contrast objective. Fluorescence images were obtained with a Hamamatsu ORCA-R^2^ digital charge-coupled-device camera, and the light source was the Xcite 120 light-emitting diode (Lumen Dynamics, Mississauga, Ontario, Canada). Emission filters were purchased from Chroma Technology (Bellows Falls, VT). Specific emission filters were DAPI-5060C-OMF (excitation [EX] filter, 377/50 nm; emission [EM] filter, 447/60 nm; dichroic mirror [DM], 409 nm), GFP-3035D-OMF (EX filter, 473/31 nm; EM filter, 520/35 nm; DM, 495 nm), mCherry-B-OFF (EX filter, 562/40 nm; EM filter, 641/75 nm; DM, 593 nm). Images were processed with the Olympus software package cellSense Dimensions (v 1.14). The *V. vulnificus* fliM-tdTomato fusion was cloned into pSU38 (27) using the Gibson assembly kit (New England Biolabs, Ipswich, MA), and expression was induced with 0.1% l-Ara (Sigma-Aldrich). Where indicated, cells were stained with DAPI for 5 min prior to the addition of 6-carboxyfluorescein (FAM)−SMAP-29D.

### Microfluidics.

Imaging of individual cells was conducted at 30°C using the CellAsic microfluidic perfusion system (ONIX) with integrated temperature controller and B04A bacterial microfluidic plates (EMD Millipore, Billerica, MA). The CellAsic ONIX FG software (v 5.0.2.0) was used to control flow rate and deliver fresh medium with or without 100 µM ampicillin or SMAP-29. Chambers were loaded by perfusion of 50 µl of a 10^6^ cells/ml bacterial suspension at 2 lb/in^2^ for 15 s to prime the cells followed by 4 lb/in^2^ for 15 s to trap the cells. The chamber was then rinsed at 1 lb/in^2^ for 30 s followed by 5 lb/in^2^ for 5 min. To quickly switch solutions, media containing antibiotic or peptide were perfused at 10 lb/in^2^ for 10 s, and then flow was reduced to 2 lb/in^2^ for the remainder of the imaging.

## SUPPLEMENTAL MATERIAL

Table S1  Bactericidal activity of 1 mM SMAP-29D and BMAP-27B on colony formation by several species of bacteria. Table S1, PDF file, 0.1 MB

Figure S1  The peptides that are unable to enhance TLR3 and TLR9 signaling are also decreased for association of the FRPL-1 receptor that promotes nucleic acid-peptide endocytosis. (A) Quantification of the reduction in the mRNA levels of three known receptors that interact with LL-37 after siRNA knockdown. BEAS-2B cells were transfected with siRNA at 30 nM for 48 h, followed by extraction of the mRNAs and reverse transcription-PCR (RT-PCR) to quantify the individual RNAs. The constitutively expressed glyceraldehyde-3-phosphate dehydrogenase (GAPDH) from each sample was also quantified to allow normalization of the RNAs. (B) The peptides that retain partial enhancement of TLR3 signaling (RL-37 and CAP-11) require the FPRL-1 receptor. The mock samples were not treated with the dsRNA mimic, poly(I⋅C), or peptide. Download Figure S1, PDF file, 0.05 MB

Figure S2  Effects of cathelicidins on cell proliferation. (A) Effects of a panel of cathelicidins on the proliferation of human lung epithelial BEAS-2B cells. Cell proliferation was assessed by the 3-(4,5-dimethylthiazol-2-yl)-2,5-diphenyltetrazolium bromide (MTT) assays (top) and the Wst-1 assays (bottom). All cathelicidins were added to the cells at a final concentration of 2 µM for 3 h prior to the assessment of their effects on mitochondrial oxidoreductase, and mitochondrial dehydrogenase activities were measured in a plate reader. The data are plotted as the ratio of the samples treated with peptides to the mock-treated samples. All data were analyzed in triplicate. (B) Derivatives of SMAP-29 can have reduced negative effects on cell proliferation. (C) Derivatives of BMAP-27 can have reduced negative effects on cell proliferation. Download Figure S2, PDF file, 0.1 MB

Figure S3  Effects of cathelicidins on Toll-like receptor signaling. (A) Nonhuman cathelicidins and their derivatives have reduced enhancement of TLR3 signaling. TLR3 signaling was assessed by quantifying IL-6 levels secreted by BEAS-2B cells using ELISAs. The cells were treated with poly(I⋅C) at 0.13 µg/ml. (B) Nonhuman cathelicidins and their derivatives retain the suppression of TLR4 signaling. TLR4 signaling was assessed by quantifying IL-6 levels secreted by BEAS-2B cells using ELISAs. The cells were treated with poly(I⋅C) at 0.5 µg/ml. Download Figure S3, PDF file, 0.1 MB

Movie S1A  Time-lapse flow cell visualization of bacterial killing of *V. vulnificus* cells cultured in LB. The SMAP-29D peptide covalently labeled with fluorescein is shown in green, and DNA stained with 4′,6-diamidino-2-phenyllindole (DAPI) is shown in blue. Download Movie S1A, MOV file, 3.9 MB

Movie S1B  Time-lapse flow cell visualization of *V. vulnificus* cell following influx of ampicillin to a final concentration of 100 µM. The SMAP-29D peptide covalently labeled with fluorescein is shown in green, and DNA stained with 4′,6-diamidino-2-phenyllindole (DAPI) is shown in blue. Download Movie S1B, MOV file, 11.3 MB

Movie S1C  Time-lapse flow cell visualization of *V. vulnificus* cell following influx of SMAP-29D to a final concentration of 2 µM. The SMAP-29D peptide covalently labeled with fluorescein is shown in green, and DNA stained with 4′,6-diamidino-2-phenyllindole (DAPI) is shown in blue. Download Movie S1C, MOV file, 5 MB

Movie S1D  Time-lapse flow cell visualization of displacement of *V. vulnificus* cellular contents into the SMAP-29D-generated lesions. The SMAP-29D peptide covalently labeled with fluorescein is shown in green, and DNA stained with 4′,6-diamidino-2-phenyllindole (DAPI) is shown in blue. Download Movie S1D, MOV file, 0.1 MB
