# Supplementary material for: Cathelicidin Antimicrobial Peptides with Reduced Activation of Toll-Like Receptor Signaling Have Potent Bactericidal Activity against Colistin-Resistant Bacteria
Source: mBio. 2016 Sep 20;7(5):e01418-16. doi: 10.1128/mBio.01418-16 (PMC5030359; doi:10.1128/mBio.01418-16)
Supplement: Table S1 — Bactericidal activity of 1 mM SMAP-29D and BMAP-27B on colony formation by several species of bacteria. [file mbo004162988st1.pdf]

Supplemental Table 1. Bactericidal activity of 1 μM SMAP-29D and BMAP-27B on colony formation by several species of bacteria.

Incubation time:	0 min	5 min		30 min	
**Species**	**CFU/ml (SD)**	**CFU/ml (SD)**	**Fold redu. from T_o**	**CFU/ml (SD)**	**Fold redu. from T_o**
**SMAP-29D (2 μM)**					
En. cloacae 4080	2.4 x 10^5 (2.3 x 10^4)				
E. coli IU-342	1.0 x 10^6 (1.1 x 10^4)	2.9 x10^4 (5.9 x 10^3)	87	5.3 x10^2 (4.1 x 10^1)	453
K. pneumonia 8893	1.1 x 10^6 (2.8 x 10^4)	6.4 x 10^3 (8.5 x 10^2)	156	3.3 x10^3 (1.4 x 10^2)	303
*K. pneu*monia C-2	0.96 x 10^5 (9.6 x 10^3)	9.4 x 10^3 (9.9 x 10^2)	117	3.5 x 10^2 (7.0 x 10^1)	3142
V. fischeri	8.3 x 10^5 (7.2 x 10^4)	1.7 x 10^4 (2.1 x 10^3)	56	1.2 x 10^3 (7.0 x 10^1)	800
V. angustum	1.9 x 10^6 (8.3 x 10^5)	NT		<20 (1)	>41,500
		NT		1.0 x 10^2 (34)	>18,500
**BMAP-27B (2 μM)**					
En. cloacae 4080	4.8 x 10^5 (2.4 x 10^4)	< 200	2400	< 25	>19,200
E. coli IU-342	1.7 x 10^5 (5.6 x 10^3)	< 200	850	< 25	> 6,800
K. pneumonia 8893	2.1 x 10^5 (2.8 x 10^4)	< 200	1050	< 25	> 8,400
*K. pneu*monia C-2	6.5 x 10^5 (2.3 x 10^4)	2.6 x10^4 (5.7 x 10^3)	25	50	> 13,000
V. fischeri	9.6 x 10^5 (8.0 x 10^4)	NT		<20	> 48,000
V. angustum	2.3 x 10^6 (3.9 x 10^5)	NT		27	> 88,460
