# Supplementary material for: Cathelicidin Antimicrobial Peptides with Reduced Activation of Toll-Like Receptor Signaling Have Potent Bactericidal Activity against Colistin-Resistant Bacteria
Source: mBio. 2016 Sep 20;7(5):e01418-16. doi: 10.1128/mBio.01418-16 (PMC5030359; doi:10.1128/mBio.01418-16)
Supplement: Figure S1 — The peptides that are unable to enhance TLR3 and TLR9 signaling are also decreased for association of the FRPL-1 receptor that promotes nucleic acid-peptide endocytosis. (A) Quantification of the reduction in the mRNA levels of three known receptors that interact with LL-37 after siRNA knockdown. BEAS-2B cells were transfected with siRNA at 30 nM for 48 h, followed by extraction of the mRNAs and reverse transcription-PCR (RT-PCR) to quantify the individual RNAs. The constitutively expressed glyceraldehyde-3-phosphate dehydrogenase (GAPDH) from each sample was also quantified to allow normalization of the RNAs. (B) The peptides that retain partial enhancement of TLR3 signaling (RL-37 and CAP-11) require the FPRL-1 receptor. The mock samples were not treated with the dsRNA mimic, poly(I⋅C), or peptide. Download [file mbo004162988sf1.pdf]

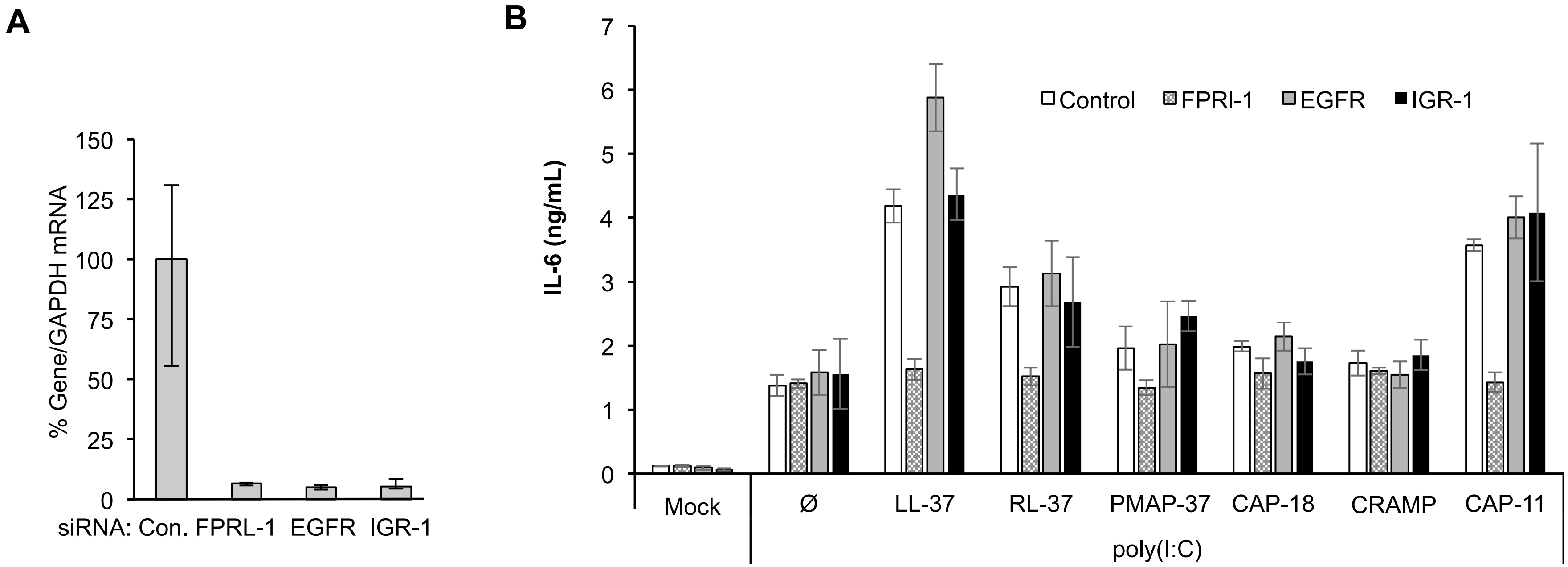

Supplemental Figure 1. The peptides that are unable to enhance TLR3 and TLR9 signaling are also decreased for association of the FRPL-1 receptor that promotes nucleic acid-peptide endocytosis. **A**) Quantification of the reduction in the mRNA levels of three known receptors that interact with LL-37 after siRNA knockdown. BEAS-2B cells were transfected with siRNA at 0.3 nM for 48 h, followed by extraction of the mRNAs and RT-PCR to quantify the individual RNAs. The constitutively expressed GAPDH from each sample was also quantified to allow normalization of the RNAs. **B**) The peptides that retain partial enhancement of TLR3 signaling (RL-37 and CAP-11) require the FPRL-1 receptor. The mock samples were not treated with the dsRNA mimic, poly(I:C) or peptide.
