# Supplementary material for: Cathelicidin Antimicrobial Peptides with Reduced Activation of Toll-Like Receptor Signaling Have Potent Bactericidal Activity against Colistin-Resistant Bacteria
Source: mBio. 2016 Sep 20;7(5):e01418-16. doi: 10.1128/mBio.01418-16 (PMC5030359; doi:10.1128/mBio.01418-16)
Supplement: Figure S2 — Effects of cathelicidins on cell proliferation. (A) Effects of a panel of cathelicidins on the proliferation of human lung epithelial BEAS-2B cells. Cell proliferation was assessed by the 3-(4,5-dimethylthiazol-2-yl)-2,5-diphenyltetrazolium bromide (MTT) assays (top) and the Wst-1 assays (bottom). All cathelicidins were added to the cells at a final concentration of 2 µM for 3 h prior to the assessment of their effects on mitochondrial oxidoreductase, and mitochondrial dehydrogenase activities were measured in a plate reader. The data are plotted as the ratio of the samples treated with peptides to the mock-treated samples. All data were analyzed in triplicate. (B) Derivatives of SMAP-29 can have reduced negative effects on cell proliferation. (C) Derivatives of BMAP-27 can have reduced negative effects on cell proliferation. Download [file mbo004162988sf2.pdf]

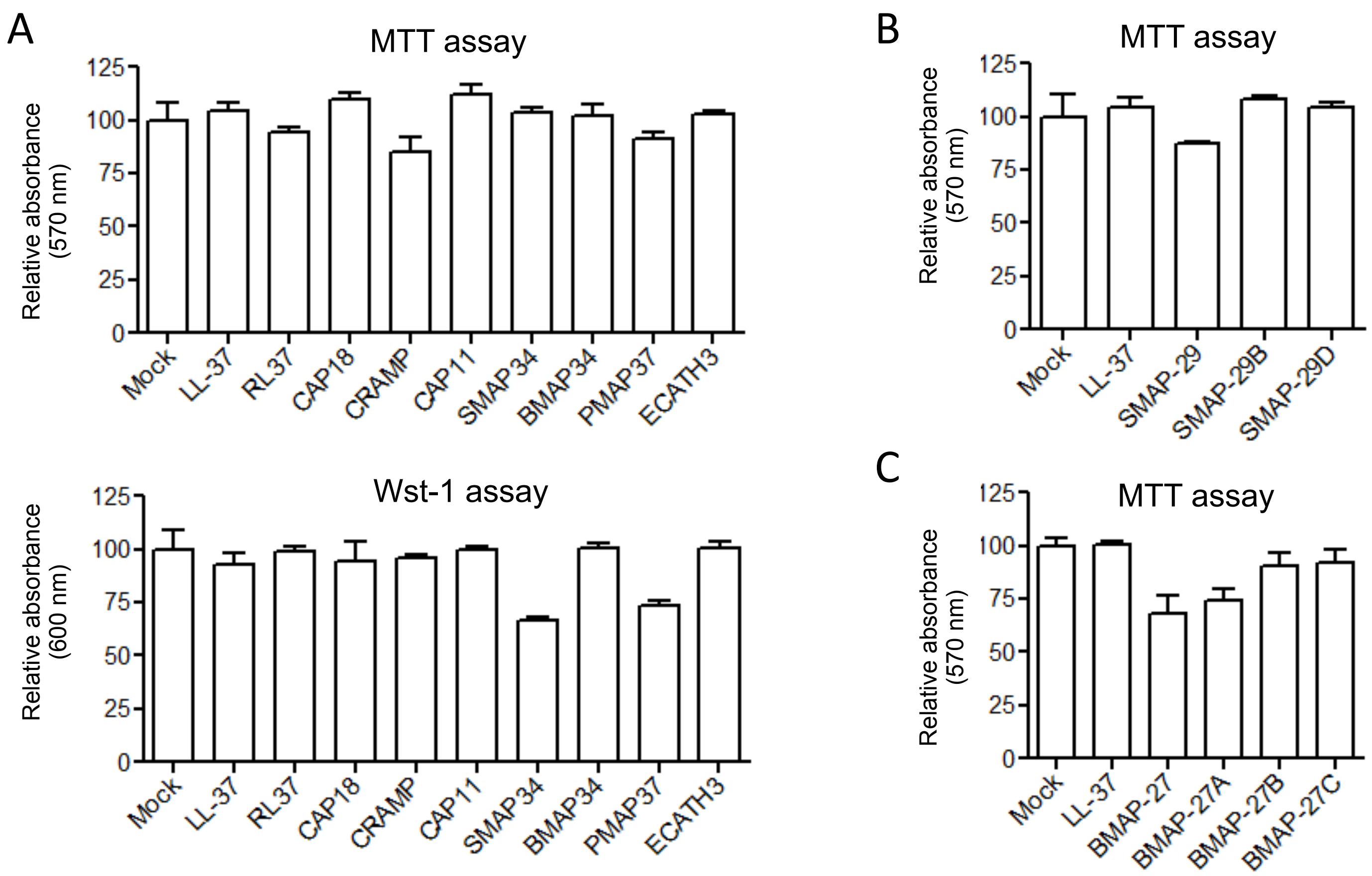

Supplemental Figure 2. Effects of cathelicidins on cell proliferation. **A**) Effects of a panel of cathelicidins on the proliferation of human lung epithelial BEAS-2B cells. Cell proliferation was assessed by the MTT assays (top) and the Wst-1 assays (bottom). All cathelicidins were added to the cells at 2 μM final concentration for 3 h prior to the assessment of their effects on mitochondrial oxidoreductase and mitochondrial dehydrogenase activities were measures in a plate reader. The data is plotted as the ratio of the samples treated with peptides to the mock-treated samples. All data were analyzed in triplicates. **B**) Derivatives of SMAP-29 can have reduced negative effects on cell proliferation. **C**) Derivatives of BMAP-27 can have reduced negative effects on cell proliferation.
