# Supplementary material for: Cathelicidin Antimicrobial Peptides with Reduced Activation of Toll-Like Receptor Signaling Have Potent Bactericidal Activity against Colistin-Resistant Bacteria
Source: mBio. 2016 Sep 20;7(5):e01418-16. doi: 10.1128/mBio.01418-16 (PMC5030359; doi:10.1128/mBio.01418-16)
Supplement: Figure S3 — Effects of cathelicidins on Toll-like receptor signaling. (A) Nonhuman cathelicidins and their derivatives have reduced enhancement of TLR3 signaling. TLR3 signaling was assessed by quantifying IL-6 levels secreted by BEAS-2B cells using ELISAs. The cells were treated with poly(I⋅C) at 0.13 µg/ml. (B) Nonhuman cathelicidins and their derivatives retain the suppression of TLR4 signaling. TLR4 signaling was assessed by quantifying IL-6 levels secreted by BEAS-2B cells using ELISAs. The cells were treated with poly(I⋅C) at 0.5 µg/ml. Download [file mbo004162988sf3.pdf]

A

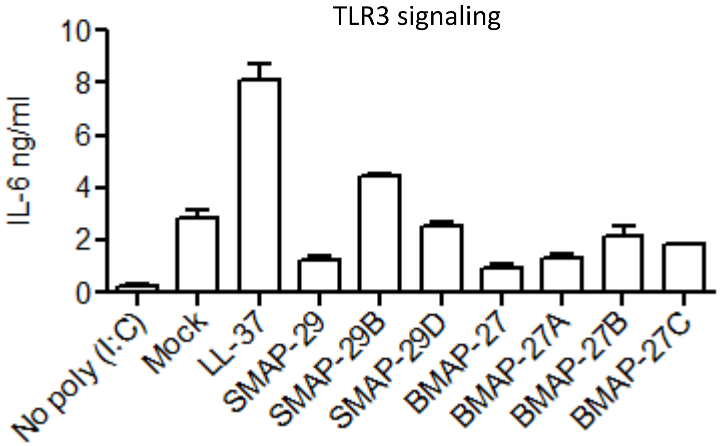

B

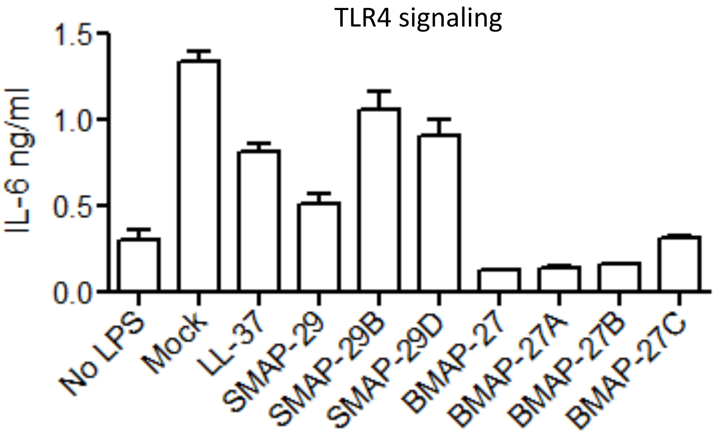

Supplemental Figure 3. Effects of cathelicidins on Toll-like receptor signaling. **A**) Nonhuman cathelicidins and their derivatives have reduced enhancement of TLR3 signaling. TLR3 signaling was assessed by quantifying IL-6 levels secreted by BEAS-2B cells using ELISAs. The cells were treated with poly(I:C) at 0.13 μg/ml. **B**) Non-human cathlicidins and their derivatives retain the suppression of TLR4 signaling. TLR4 signaling was assessed by quantifying IL-6 levels secreted by BEAS-2B cells using ELISAs. The cells were treated with poly(I:C) at 0.5 μg/ml.
